# Supplementary material for: Towards population genomics in non-model species with large genomes: a case study of the marine zooplankton Calanus finmarchicus
Source: R Soc Open Sci. 2019 Feb 13;6(2):180608. doi: 10.1098/rsos.180608 (PMC6408391; doi:10.1098/rsos.180608)
Supplement: Electronic Supplementary Materials [file rsos180608supp1.pdf]

Electronic Supplementary Material

From: **Towards population genomics in non-model species with large genomes: a case study of the marine zooplankton *Calanus finmarchicus***

Marvin Choquet[1*] - Irina Smolina[1], Anusha K. S. Dhanasiri[1], Leocadio Blanco-Bercial[2], Martina Kopp[1], Alexander Jueterbock[1], Arvind Y. M. Sundaram[3], Galice Hoarau[1]

(corresponding author)*

[1] Faculty of Biosciences and Aquaculture, Nord University, Bodø, Norway

[2] Bermuda Institute of Ocean Sciences, St George's, Bermuda

[3] Norwegian Sequencing Centre, Department of Medical Genetics, Oslo University Hospital, Oslo, Norway

Content:

Supplementary Material 1:

Pilot study: ddRAD-seq applied to *Calanus finmarchicus*

Supplementary Material 2:

Sequence capture-enrichment detailed protocols and complementary analyses

Supplementary Material 3:

Custom-made scripts for sequence capture-enrichment data analyses in *Calanus finmarchicus* and *C. glacialis*

References of the Supplementary Material

Supplementary Material 1:

Pilot study: ddRAD-seq applied to *Calanus finmarchicus*

Materials and methods

1- Samples and DNA extraction

For the ddRAD-seq approach, zooplankton was sampled from 6 locations that span the distributional range of *Calanus finmarchicus* (Supp. Table 1). Samples were collected by vertical tows between either 0-100 m or 0-200 m depth using WP2 [1] or similar nets with mesh size of 200 µm and immediately preserved in 95 % undenatured ethanol, with subsequent change of ethanol after 24 h. Total genomic DNA was extracted individually using the E.Z.N.A. Insect DNA Kit (Omega Bio-Tek) or E.Z.N.A. DNA/RNA Isolation Kit (Omega Bio-Tek) according to manufacturers' instructions. *Calanus* species identification was performed using a set of six nuclear insertion-deletion markers (InDels) [2].

Supp. Table 1: *Calanus finmarchicus* sample information.

Location	n	Collection date	Lat.	Long.
West Greenland (WG)	16	25.05.2012	69.23 N	-53.38 E
East Greenland (EG)	16	10.08.2012	74.31 N	-20.25 E
Barents Sea (BAR)	16	06.08.2012	70.50 N	19.99 E
Norwegian Sea (N12)	16	03.08.2012	64.67 N	0.00 E
Norwegian Sea (N13)	16	05.05.2013	65.05 N	-0.86 E
Bay St. Lawrence (STL)	16	17.04.2013	47.27 N	-59.80 E

2- Library preparation and sequencing

Sixteen individuals of *C. finmarchicus* per location were used for analyses (Supp. Table 1), more than is necessary (n ≤ 6) to estimate genetic differentiation with > 100 markers [3]. Pool-ddRAD-seq libraries were prepared according to Peterson *et al.* (2012)[4], with minor modifications. We aimed for 8,000 fragments per pool, covered 200 times. Two restriction enzymes were selected based on *in silico* digestion of a previously-sequenced portion of the *C. finmarchicus* genome (≈ 0.5%) [2] using the R package SimRAD (version 0.96) [5]. Among six commonly used restriction enzymes (*Msp*I, *Bam*HI, *Eco*RI, *Sbf*I, *Sph*I, *Nla*III, *Mlu*CI), the pair *Sbf*I-*Eco*RI resulted in a suitable number of fragments in the 400-500 bp size range. DNA from the 16 individuals from each sampling location was pooled in equal amounts. The six pools of approximately 100 ng were individually digested overnight at 37°C with 20,000 units of *Eco*RI-HF (New England BioLabs) and *Sbf*I-HF (New England BioLabs) enzymes in CutSmart buffer (New England BioLabs) with total volume of 50 µl. Reactions were cleaned with the Agencourt AMPure XP system (Beckman Coulter) using 1.5x volume of the AMPure reagent. Digested DNA fragments were ligated with adapters for 10 min at room temperature using the Quick Ligation Kit (New England BioLabs). The ligation products were cleaned with Agencourt AMPure XP system (Beckman Coulter), as described above. Ligated and cleaned fragments ranging between 480–580 bp (length of adapters was taken into account) were selected separately for each library using a 2% agarose gel E-Gel SizeSelect (Invitrogen). All obtained DNA was amplified by 15 cycles of Polymerase Chain Reaction (PCR – with annealing temperature at 62°C) in a total volume of 50 µl using the Phusion High-Fidelity PCR kit (New England BioLabs) and according to the manufacturer´s instructions. Reactions were cleaned with the Agencourt AMPure XP system (Beckman Coulter) using 0.8x volume of the reagent. Products were size selected using a 2% agarose gel E-Gel SizeSelect (Invitrogen) and ran on the Agilent 2200 TapeStation System (Agilent Technologies) for quantification. Libraries were pooled in equal amounts and sequenced with MiSeq Reagent Kits v2 on a 500 cycles chip (Illumina).

3- SNP genotyping

The sequenced reads were assigned to their corresponding pool (associated to their location) identified by six barcodes using *DDemux* [6]. Adapter and quality trimming was performed using Trim Galore! (Babraham Bioinformatics – version 0.4.2), with Phred quality > 20 and length > 20 bp. As there is no reference genome for *Calanus*, trimmed reads were *de novo* assembled with the ddRAD assembler Rainbow v2.0.3 [7] and clustered with CD-HIT [8], as implemented in the dDocent pipeline (version 2.2.16) for ddRAD population genomics [9].

Specificity of Pool-seq data was taken into account in further analyses, as recommended in Schlötterer et al. (2014)[10]. Cleaned reads were mapped to the *de novo* assembly using Bowtie2 v2.2.3 [11], with the following parameters: end-to-end -I 40 -X 850 -N 1 -L 20 -D 20 -R 3 -i S,1,0.50. Only pairs of reads that were uniquely and concordantly mapped with quality ≥ 20 were selected for further analysis using a custom-made script. To avoid false-positive SNPs, reads were realigned around InDels using the Genome Analysis Toolkit v.3 [12], as described in Wit et al. (2012) [13]. SNPs were called on a merged bam file containing aligned reads of all six locations with SNVerGUI (version 0.5.3) [14], which calls single nucleotide variants from pooled data and evaluates the significance of a candidate locus to be a variant. SNPs were filtered from obtained polymorphic sites with the following settings: minimum number of alleles = 2 and minimum coverage = 96 per pool (i.e., 6x per individual) using VCFtools (version 0.1.13) [15]. Only high quality SNPs were kept and the regions with coverage > 1,000 were excluded, considering that they may represent clusters of multi-copy genetic regions that can inflate the number of false-positive SNPs.

Results

On average, 99.85 % of the reads passed quality filtering. *De novo* assembly resulted in 41,500 contigs covering 17,886,794 bp with a mean GC content of 38.9 %. The contigs were on average 430.9 bp long (259 – 758 bp). However, when the forward and reverse reads did not overlap, they were connected with up to 10 “N” bases. In total 2,836 contigs were annotated (6.83 %). Most (87.23 %) of the reads mapped concordantly and with a mapping quality > 20. However, on average, only 21.76 % of the reads mapped uniquely (Supp. Table 2), resulting in the mean coverage per location library of 16x (Supp. Fig. 1a).

Overall, in all six libraries (corresponding to six pools of *C. finmarchicus* individuals from six different locations), 24,701 single nucleotide variants were detected with SNVerGUI software. Among these variants, 15,285 were high-quality SNPs, but only 1,871 SNPs were covered > 96x per library (Supp. Fig. 1b). Within maximum (1000x) and minimum (96x) coverage thresholds, the average number of SNPs per location was 510, with a minimum of 211 in Bay St. Lawrence (due to fewer sequenced reads) and a maximum of 625 in the Norwegian Sea (location N12). The maximum number of SNPs on the same contig was 14 (Supp. Fig. 2). On average, the SNPs were distributed over 99 contigs (Supp. Table 2) per library. A total of 343 SNPs, located over 32 contigs, were found to be common among all six locations.

Supp. Table 2. Summary of sequenced reads and discovered SNPs using ddRAD at six locations for *Calanus finmarchicus*. Raw reads: total sequenced reads; % clean reads: reads without adapters, a Phred quality > 20, and length > 20 bp; % HQ-mapped reads: reads that mapped to a unique site in the genome reference with a Phred quality > 20 and in proper pairs; NCBI BioSample accessions: accession numbers to fastq files of raw reads.

Location	Raw reads	% clean reads	% HQ-mapped reads	Total # SNPs	Unique SNPs	Contigs with SNPs	NCBI BioSample accessions
WG	1,369,668	99.85 %	19.44 %	570	147	96	SAMN04296798
EG	1,362,884	99.87 %	22.23 %	559	148	116	SAMN04296799
BAR	1,296,722	99.86 %	21.44 %	565	162	109	SAMN04296800
N12	1,562,076	99.83 %	23.80 %	625	193	124	SAMN04296801
N13	1,117,632	99.85 %	24.02 %	535	165	106	SAMN04296802
STL	617,428	99.80 %	19.62 %	211	34	43	SAMN04296803

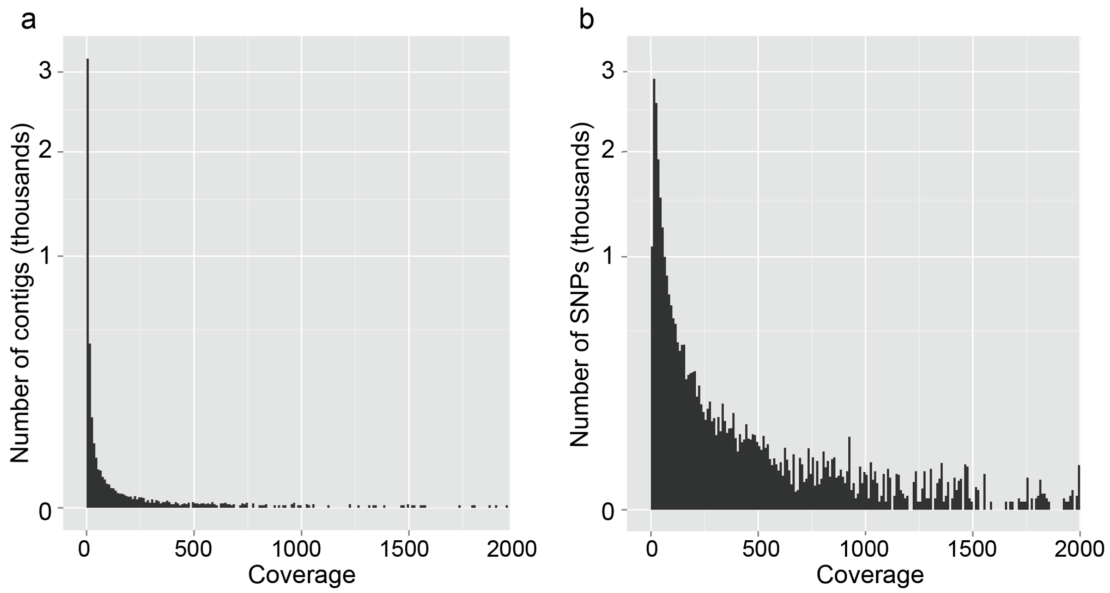

Supp. Fig. 1. Number of contigs (a) and SNPs (b) in relation to depth of coverage.

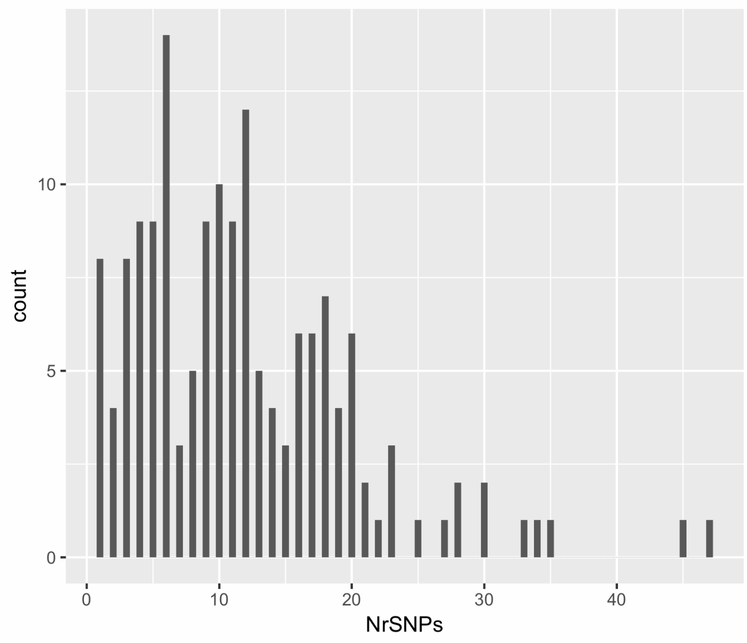

Supp. Fig. 2. Frequency of contigs according to number of SNPs per contig obtained from Pool-ddRAD-seq in *Calanus finmarchicus*.

Discussion

Our pilot study using Pool-ddRAD-seq data and strict quality filtering resulted in an average of 510 SNPs per location, with 343 SNPs in common among all locations. This is much lower than for most RAD-seq or Pool-seq studies that generally yield thousands of SNPs (e.g. [16-19]) and is likely due to the low coverage of ddRAD contigs (mean coverage was 16x per pool) that does not allow discriminating real SNPs from amplification or sequencing errors. The low contig coverage is likely due to the *in silico* under-estimation of ddRAD fragments and low success (28 %) of unique and high-quality mapping, which are related to the large *C. finmarchicus* genome. Further, mapping reads from the pooled samples is challenging, since the population pool may be highly polymorphic; however, too-liberal mapping parameters increase the chances of incorrect mapping [20]. Therefore, we followed recommendations for the pooled data [10, 20], resulting in fewer SNPs, but higher confidence levels.

Most commonly, species that are analysed using RAD- or ddRAD-seq have genome sizes < 5 Gb, resulting in high numbers of usable fragments and SNPs (e.g. [17, 21, 22]). While few studies have investigated species with large genomes for applications other than marker development, these serve to highlight the difficulties (e.g. [23]). Initially, ddRAD-seq was designed to allow more flexible control over the number of obtained

contigs and can result in several orders of magnitude variation in the number of fragments by using restriction enzymes and selecting fragments of specific sizes [4, 24], which benefits the analysis of species with large genomes. However, it can be difficult to select the enzyme pair in uncharacterized genomes with unknown frequencies of the restriction sites [21]. Furthermore, if a small fraction of a species' genome is known, it may not be representative of the entire genome. In the present study, the selected enzyme pair resulted in 5 times more ddRAD contigs than was expected, and consequently reduced the average coverage per contig and the number of contigs that were of sufficient quality for further analysis. Overall, this study and other recent studies that applied RAD methods for non-model species with large genomes (e.g. [17, 23]) suggest that significant challenges remain for both, Pool-seq and (dd)RAD-seq, for species with relatively large genomes (> 5 Gb).

Supplementary Material 2:

Sequence capture-enrichment detailed protocols and complementary analyses

Transcriptome-based capture:

Library preparation & capture

The DNA (100 ng) was fragmented to an average size of 500 bp by sonication using a Covaris shearing instrument. Fragmented DNA was subsequently end-repaired and A-tailed using the KAPA library preparation kit (Kapa Biosystems) following the producer's recommendations. The index adapter (SeqCap Adapter Kit, Roche NimbleGen) was ligated at 16°C overnight. A size-selection for > 450 bp fragments was performed using AMPure XP beads (Beckman Coulter). Further, fragments were amplified by PCR for 7 cycles using KAPA HiFi HotStart ReadyMix (Kapa Biosystems) and cleaned-up with AMPure XP beads (Beckman Coulter). A final size selection of fragments with lengths between 450 and 700 bp was performed using Pippin Prep (Sage Science). Hybridisation of the sample to the probes was performed overnight, with the SeqCap Hybridisation and Wash Kit (Roche NimbleGen). Capture of the DNA by streptadivin-coated magnetic beads was done over 45 minutes in a thermocycler at 47°C. The captured sequences were cleaned up (AMPure XP beads – Beckman Coulter) and then amplified by PCR for 10 cycles. The resulting amplified sequences were cleaned up using AMPure XP beads (Beckman Coulter). Sequencing was carried out on a MiSeq sequencer (Illumina) with 2x300bp v.3 chemistry.

Genome-based capture:

Library preparation & capture

All DNA libraries were fragmented to an average size of 300 bp by sonication (starting DNA amount = 60 ng for each *C. finmarchicus* libraries and 130 ng for each *C. glacialis* libraries), and were prepared using the NEXTflex™ Rapid Pre-Capture Combo Kit (Bioo Scientific, Austin, TX, USA), including a step of single adapter indexing of each library. Libraries were cleaned-up, amplified separately for 7 cycles of PCR and pooled per species.

We increased the efficiency of the hybridisation and aimed to maximise the number of on-target captured sequences by doing the capture reaction twice, splitting the total amount of baits required for one reaction in two. The first round of hybridisation was performed in parallel for the two species/pools, using 4 µL of baits for each reaction.

The reaction was performed over three days at a temperature of 60°C in order to maximise the specificity. Capture was performed consecutively using DYNAbeads MyOne Streptavidin C1 beads (Invitrogen) to bind the hybridised targets during 30 min at 65°C. The captured DNA was amplified by PCR for 8 cycles using KAPA HiFi HotStart ReadyMix (Kapa Biosystems). A second round of hybridisation was conducted using 1.5 µL of baits for each of the two pools, followed by a second capture and 6 more cycles of post-capture PCR. Finally, the two pools were mixed together in equal proportions and sequenced on a NextSeq 550 (Illumina) with a 2x150 bp mid-output kit v.2.

Genome-based capture efficiency – Complementary results:

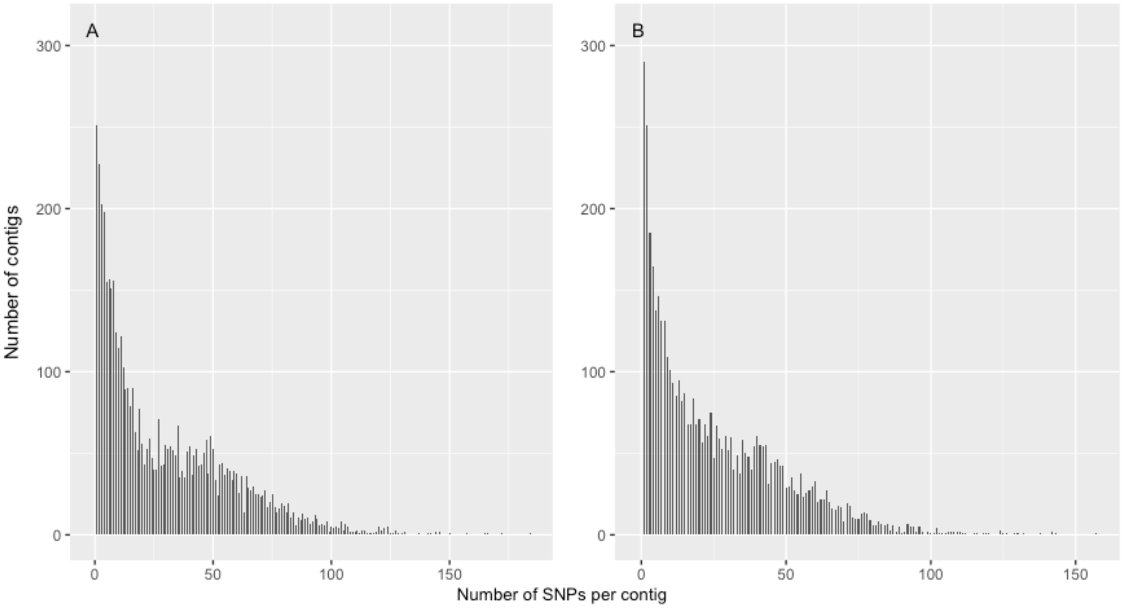

Supp. Fig. 3. **Distribution of filtered SNPs per contig obtained from capture enrichment.** (**A**) In *Calanus finmarchicus*, 154,087 SNPs were identified on 4,603 contigs. (**B**) In *C. glacialis*, 121,872 SNPs were identified on 5,363 contigs. Only the SNPs hard-filtered, phased and well covered are represented.

Investigation for loci under selection:

Site Frequency Spectrum

For *C. finmarchicus*, individuals from all 3 locations showed very similar frequencies. The drop of frequencies between 0.25 and 0.35 is also due to the low number of individuals and binning: meanwhile having 4 alleles of the 16 (8 individuals, 2 alleles each) is 0.25, 5 of 16 corresponds to a frequency of 0.3125 (0.35 bin). The few cases of alleles with frequencies with $0.25 < x \leq 0.3$ are those loci that were missing in one individual in the population (due to the 80% constrain; ~20% of the SNP sites), therefore the derived frequencies are slightly different.

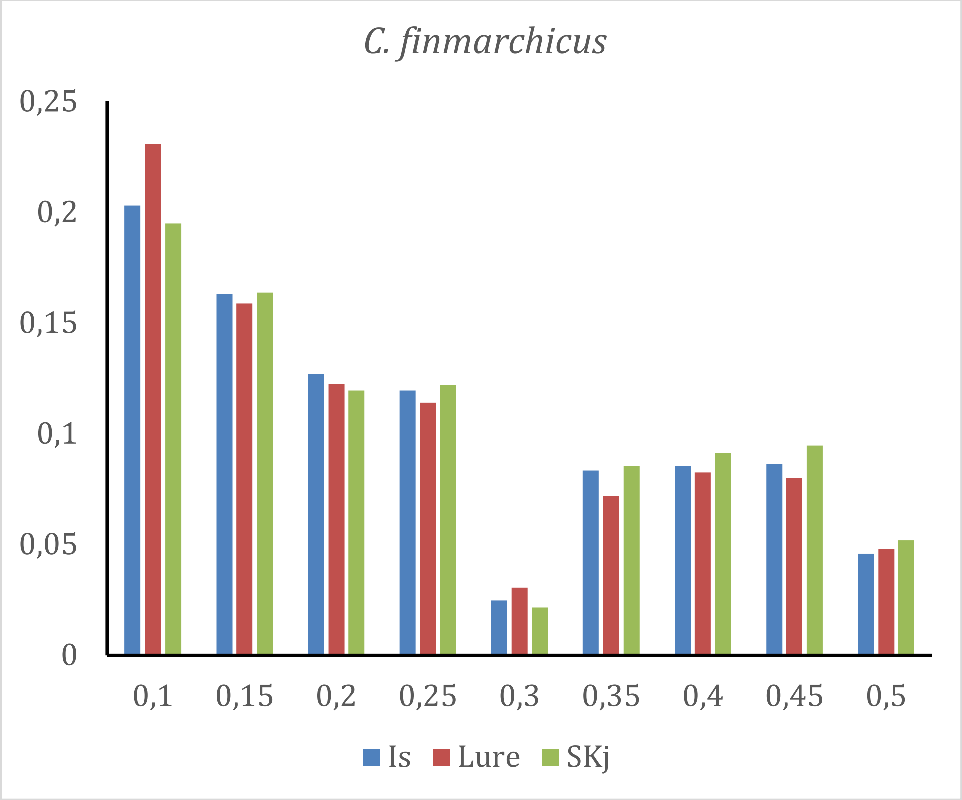

Supp. Fig. 4. Site frequency spectrum of all SNPs per locations in *Calanus finmarchicus*. Only SNPs with > 5x coverage and present in more than 80% of the individuals were used (= 46,544 SNPs). The x axis corresponds to the frequencies representing the number of loci for each minor allele frequency within each location (Is: Isfjord; Lure: Lurefjord; Skj: Skjerstadfjord). The y axis corresponds to the proportion of the frequency within each location.

For *C. glacialis*, there was a difference between the Isfjord and the Lurefjord, and the Skjerstadfjord, due to the different number of individuals from the Skjerstadfjord (3 and 3, and 6, respectively). This fact makes that the minimum MAF (minor allele frequency) corresponded to 1/6 for the Isfjord and the Lurefjord, and 1/12 for the Skjerstadfjord. The low number of individuals also caused the very low number of populated categories in the histogram. Again, some poorly populated intermediate categories are derived from loci that missed 1 individual at that population (with 80% coverage, one individual was allowed to be missed). Since the Skjerstadfjord had more individuals, categories are more homogenously distributed.

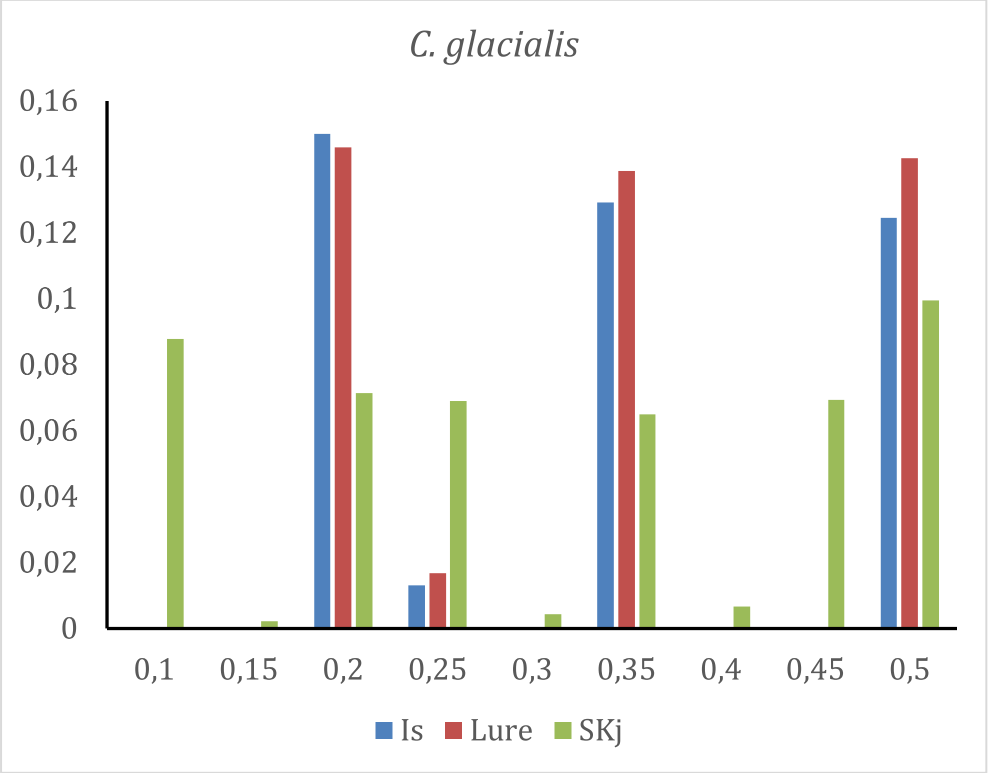

Supp. Fig. 5. Site frequency spectrum of all SNPs per locations in *Calanus glacialis*. Only SNPs with > 5x coverage and present in more than 80% of the individuals were used (= 49,742 SNPs). The x axis corresponds to the frequencies representing the number of loci for each minor allele frequency within each location (Is: Isfjord; Lure: Lurefjord; Skj: Skjerstadfjord). The y axis corresponds to the proportion of the frequency within each location.

Supplementary Material 3:

Custom-made scripts for sequence capture-enrichment data analyses in *Calanus finmarchicus* and *C. glacialis*

The scripts displayed here contain commands that were used for sequence capture data analyses from the step of mapping the raw sequencing reads until the step of phasing the hard-filtered SNPs.

```
### Example of analyses conducted for the individual named "CF_Is_1"
### Mapping raw reads directly to the assembly of contigs “Masurca Assembly”:
> bwa mem -M MasurcaAssembly CF_Is_1_R1.fastq CF_Is_1_R2.fastq | samtools view\
> -Sbh -o CF_Is_1_aln.bam \

### Filtering to keep only reads concordantly mapped, in pairs, and uniquely:
> samtools view CF_Is_1_aln.bam | fgrep XA | cut -f 1 > bad_names.txt
> samtools view -h CF_Is_1_aln.bam  | fgrep -vf bad_names.txt | samtools view \
> -Sb - > CF_Is_1_aln2.bam \
> samtools view CF_Is_1_aln2.bam | fgrep SA | cut -f 1 > bad_names2.txt
> samtools view -h CF_Is_1_aln2.bam  | fgrep -vf bad_names2.txt | samtools view \
> -Sb - > CF_Is_1_aln3.bam \
> samtools view -b -F 3332 -F 4 -f 1 -f 2 CF_Is_1_aln3.bam >CF_Is_1_aln3_cleaned.bam

### Sorting of the reads by the leftmost mapping coordinates:
> samtools sort CF_Is_1_aln3_cleaned.bam -o CF_Is_1_aln3_cleaned_sorted.bam

### Mark duplicates with Picard tools and remove them:
> java -jar picard.jar MarkDuplicates INPUT=CF_Is_1_aln3_cleaned_sorted.bam \
> OUTPUT=CF_Is_1_dedup.bam METRICS_FILE=CF_Is_1_dedup_metricsfile \
> ASSUME_SORTED=true \
> VALIDATION_STRINGENCY=SILENT REMOVE_DUPLICATES=true

### Add or replace Read Groups:
> java -jar picard.jar AddOrReplaceReadGroups I=CF_Is_1_dedup.bam \
> O=CF_Is_1_dedup_RG.bam SORT_ORDER=coordinate RGLB=lib1 RGPL=illumina \
> RGPU=unit33 RGSM=CF_Is_1 \

### Index BAM file:
> samtools index CF_Is_1_dedup_RG.bam

### Valide SAM file:
> java -jar picard.jar ValidateSamFile I=CF_Is_1_dedup_RG.bam MODE=SUMMARY

### Realignment around InDels: RealignerTargetCreator (from GATK version 3.7)
```

```
> java -jar GenomeAnalysisTK.jar -T RealignerTargetCreator -R MasurcaAssembly.fasta\
> -I CF_Is_1_dedup_RG.bam -o CF_Is_1_realigner.intervals \


### Realignment around InDels: IndelRealigner (from GATK version 3.7)
> java -jar GenomeAnalysisTK.jar -T IndelRealigner -R MasurcaAssembly.fasta \
> -I CF_Is_1_dedup_RG.bam -targetIntervals CF_Is_1_realigner.intervals \
> -o CF_Is_1_realigned.bam \

### HaplotypeCaller for Variant Discovery (from GATK version 3.7)
> java -jar GenomeAnalysisTK.jar -T HaplotypeCaller -R MasurcaAssembly.fasta \
> -I CF_Is_1_realigned.bam -I Sample2.bam -I Samplexx.bam \
> -o output.raw.snps.indels.vcf \

### Variant hard-filtering (following Best Practices from GATK)
# when training and truth sets are not available for VariantRecalibration, then we have
# to do hard-filtering
### 1- Extract SNPs from dataset:
> java -jar GenomeAnalysisTK.jar -T SelectVariants -R MasurcaAssembly.fasta \
> -V output.raw.snps.indels.vcf -selectType SNP -o raw_snps.vcf \

### 2- Determine parameters for filtering and apply them to the SNPs:
> java -jar GenomeAnalysisTK.jar -T VariantFiltration -R MasurcaAssembly.fasta \
> -V raw_snps.vcf --filterExpression "QD < 2.0 || FS > 60.0 || MQ < 50.0 || \
> MQRankSum < -5.0 || ReadPosRankSum < -5.0" --filterName "my_snp_filter" \
> -o filtered_snps.vcf \

### SNPs phasing
> java -jar GenomeAnalysisTK.jar -T ReadBackedPhasing -R MasurcaAssembly.fasta \
> -I CF_Is_1_realigned.bam -I Sample2.bam -I Samplexx.bam –variant\
> filtered_snps.vcf -o phased_SNPs.vcf \
```

Bash script: Thinning and calculating F_{ST}

Script by **Leocadio Blanco-Bercial** (leocadio_blanco@hotmail.com – ORCID ID: 0000-0003-0658-7183).

Please cite:
Choquet et al. "Towards population genomics in non-model species with large genomes: a case study of the marine zooplankton Calanus finmarchicus" Royal Society Open Science, 2019.

```
#!/bin/bash

##STEPS:
##VCFtools to prefilter
##then repeat 1000 times:
###thinner 1 SNP per contig
###then global FST, add to unique logfile, remove all intermediate files

##VCF tools filtering by whatever filters are needed
vcftools --vcf infile.vcf --out out1 --keep list.txt --remove-indels --recode --recode-INFO-all

vcftools --vcf out1.recode.vcf --out 80min --max-missing 0.8 --non-ref-ac-any 1 --recode --recode-INFO-all

##thinning and calculating FSTs for each thinning iteration for 1000 iterations
for i in {1..1000}
do

##perl https://github.com/caballero/Scripts/blob/master/rand_var_per_chr.pl usage:
##rand_var_per_chr.pl infile > outfile
perl rand_var_per_chr.pl 80min.recode.vcf > thin.vcf

##calculating the FST in PLINK
plink --vcf thin.vcf --double-id --allow-extra-chr --within withinfin.txt --fst

##Add all log files, after each iteration, into a single log to extract later the FST values
cat plink.log >> ALLFST.txt

##Cleaning before the next loop
rm plink.log
rm plink.nosex
rm thin.vcf
rm plink.fst
```

done

References of the Supplementary Material

1 Fraser, J. 1966 Zooplankton sampling. *Nature*. **211**, 915-916.
2 Smolina, I., Kollias, S., Poortvliet, M., Nielsen, T. G., Lindeque, P., Castellani, C., Moller, E. F., Blanco-Bercial, L., Hoarau, G. 2014 Genome- and transcriptome-assisted development of nuclear insertion/deletion markers for *Calanus* species (Copepoda: Calanoida) identification. *Molecular Ecology Resources*. **14**, 1072-1079. (10.1111/1755-0998.12241).
3 Willing, E.-M., Dreyer, C., Van Oosterhout, C. 2012 Estimates of genetic differentiation measured by F_{ST} do not necessarily require large sample sizes when using many SNP markers. *PLoS One*. **7**, e42649.
4 Peterson, B. K., Weber, J. N., Kay, E. H., Fisher, H. S., Hoekstra, H. E. 2012 Double digest RADseq: an inexpensive method for de novo SNP discovery and genotyping in model and non-model species. *PLoS One*. **7**, e37135. (10.1371/journal.pone.0037135).
5 Lepais, O., Weir, J. T. 2014 SimRAD: an R package for simulation - based prediction of the number of loci expected in RADseq and similar genotyping by sequencing approaches. *Molecular Ecology Resources*. **14**, 1314-1321.
6 Rašić, G., Filipović, I., Weeks, A. R., Hoffmann, A. A. 2014 Genome-wide SNPs lead to strong signals of geographic structure and relatedness patterns in the major arbovirus vector, *Aedes aegypti*. *BMC genomics*. **15**, 275.
7 Chong, Z., Ruan, J., Wu, C.-I. 2012 Rainbow: an integrated tool for efficient clustering and assembling RAD-seq reads. *Bioinformatics*. **28**, 2732-2737.
8 Fu, L., Niu, B., Zhu, Z., Wu, S., Li, W. 2012 CD-HIT: accelerated for clustering the next-generation sequencing data. *Bioinformatics*. **28**, 3150-3152.
9 Puritz, J. B., Hollenbeck, C. M., Gold, J. R. 2014 dDocent: a RADseq, variant-calling pipeline designed for population genomics of non-model organisms. *PeerJ*. **2**, e431.
10 Schlötterer, C., Tobler, R., Kofler, R., Nolte, V. 2014 Sequencing pools of individuals--mining genome-wide polymorphism data without big funding. *Nature Reviews. Genetics*. **15**, 749.
11 Langmead, B., Salzberg, S. L. 2012 Fast gapped-read alignment with Bowtie 2. *Nature Methods*. **9**, 357-359.
12 DePristo, M. A., Banks, E., Poplin, R., Garimella, K. V., Maguire, J. R., Hartl, C., Philippakis, A. A., Del Angel, G., Rivas, M. A., Hanna, M. 2011 A framework for variation discovery and genotyping using next-generation DNA sequencing data. *Nature Genetics*. **43**, 491-498.
13 Wit, P., Pespeni, M. H., Ladner, J. T., Barshis, D. J., Seneca, F., Jaris, H., Therkildsen, N. O., Morikawa, M., Palumbi, S. R. 2012 The simple fool's guide to population genomics via RNA - Seq: an introduction to high - throughput sequencing data analysis. *Molecular Ecology Resources*. **12**, 1058-1067.
14 Wang, W., Hu, W., Hou, F., Hu, P., Wei, Z. 2012 SNVerGUI: a desktop tool for variant analysis of next-generation sequencing data. *Journal of medical genetics*. jmedgenet-2012-101001.
15 Danecek, P., Auton, A., Abecasis, G., Albers, C. A., Banks, E., DePristo, M. A., Handsaker, R. E., Lunter, G., Marth, G. T., Sherry, S. T. 2011 The variant call format and VCFtools. *Bioinformatics*. **27**, 2156-2158.

16 Hohenlohe, P. A., Amish, S. J., Catchen, J. M., Allendorf, F. W., Luikart, G. 2011 Next - generation RAD sequencing identifies thousands of SNPs for assessing hybridization between rainbow and westslope cutthroat trout. *Molecular Ecology Resources*. **11**, 117-122.
17 Pukk, L., Ahmad, F., Hasan, S., Kisand, V., Gross, R., Vasemägi, A. 2015 Less is more: extreme genome complexity reduction with ddRAD using Ion Torrent semiconductor technology. *Molecular Ecology Resources*. **15**, 1145-1152.
18 Campana, M. G., Robles García, N. M., Tuross, N. 2015 America's red gold: multiple lineages of cultivated cochineal in Mexico. *Ecology and Evolution*. **5**, 607-617.
19 Reitzel, A., Herrera, S., Layden, M., Martindale, M., Shank, T. 2013 Going where traditional markers have not gone before: utility of and promise for RAD sequencing in marine invertebrate phylogeography and population genomics. *Molecular Ecology*. **22**, 2953-2970.
20 Kofler, R., Orozco-terWengel, P., De Maio, N., Pandey, R. V., Nolte, V., Futschik, A., Kosiol, C., Schlötterer, C. 2011 PoPoolation: a toolbox for population genetic analysis of next generation sequencing data from pooled individuals. *PloS One*. **6**, e15925.
21 Davey, J. W., Hohenlohe, P. A., Etter, P. D., Boone, J. Q., Catchen, J. M., Blaxter, M. L. 2011 Genome-wide genetic marker discovery and genotyping using next-generation sequencing. *Nature Reviews. Genetics*. **12**, 499.
22 DaCosta, J. M., Sorenson, M. D. 2014 Amplification biases and consistent recovery of loci in a double-digest RAD-seq protocol. *PLoS One*. **9**, e106713. (10.1371/journal.pone.0106713).
23 Deagle, B. E., Faux, C., Kawaguchi, S., Meyer, B., Jarman, S. N. 2015 Antarctic krill population genomics: apparent panmixia, but genome complexity and large population size muddy the water. *Molecular Ecology*. **24**, 4943-4959.
24 Baird, N. A., Etter, P. D., Atwood, T. S., Currey, M. C., Shiver, A. L., Lewis, Z. A., Selker, E. U., Cresko, W. A., Johnson, E. A. 2008 Rapid SNP discovery and genetic mapping using sequenced RAD markers. *PloS One*. **3**, e3376.
